# Supplementary figures and images for: NinaB and BCO Collaboratively Participate in the β-Carotene Catabolism in Crustaceans: A Case Study on Chinese Mitten Crab Eriocheir sinensis
Source: Int J Mol Sci. 2024 May 21;25(11):5592. doi: 10.3390/ijms25115592 (PMC11171921; doi:10.3390/ijms25115592)

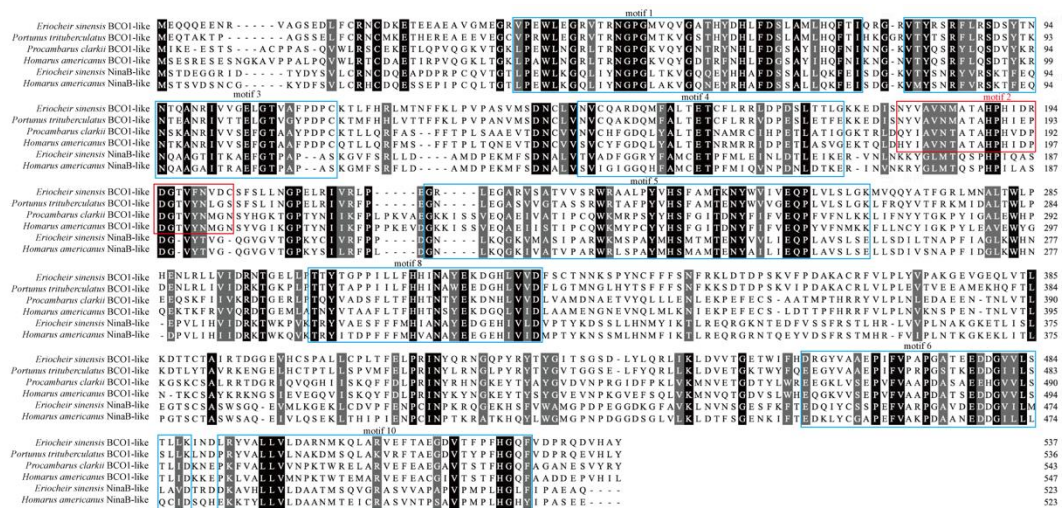

**Figure S1.** Alignment of the amino acid sequences of NinaB and BCO1

Supplement: Supplementary file 1 [file ijms-25-05592-s001.zip › ijms-2997382-supplementary.pdf]
